# Genetic Complementation of ATP Synthase Deficiency Due to Dysfunction of TMEM70 Assembly Factor in Rat

**DOI:** 10.3390/biomedicines10020276

**Published:** 2022-01-26

**Authors:** Aleksandra Marković, Kateřina Tauchmannová, Miroslava Šimáková, Petr Mlejnek, Vilma Kaplanová, Petr Pecina, Alena Pecinová, František Papoušek, František Liška, Jan Šilhavý, Jana Mikešová, Jan Neckář, Josef Houštěk, Michal Pravenec, Tomáš Mráček

**Affiliations:** 1 Institute of Physiology, Czech Academy of Sciences, Vídeňská 1083, 142 20 Prague, Czech Republic; Aleksandra.Markovic@fgu.cas.cz (A.M.); Katerina.Tauchmannova@fgu.cas.cz (K.T.); Miroslava.Simakova@fgu.cas.cz (M.Š.); Petr.Mlejnek@fgu.cas.cz (P.M.); Vilma.Kaplanova@fgu.cas.cz (V.K.); petr.pecina@fgu.cas.cz (P.P.); alena.pecinova@fgu.cas.cz (A.P.); frantisek.papousek@fgu.cas.cz (F.P.); frantisek.liska@lf1.cuni.cz (F.L.); Jan.Silhavy@fgu.cas.cz (J.Š.); jana.mikesova@uochb.cas.cz (J.M.); Jan.Neckar@fgu.cas.cz (J.N.); houstek@biomed.cas.cz (J.H.); 2 Faculty of Science, Charles University, 128 00 Prague, Czech Republic; 3 Institute of Biology and Medical Genetics, First Faculty of Medicine, Charles University and General University Hospital, 128 00 Prague, Czech Republic

**Keywords:** mitochondria disease, ATP synthase deficiency, TMEM70 factor, transgenic rescue, gene therapy

## Abstract

Mutations of the *TMEM70* gene disrupt the biogenesis of the ATP synthase and represent the most frequent cause of autosomal recessive encephalo-cardio-myopathy with neonatal onset. Patient tissues show isolated defects in the ATP synthase, leading to the impaired mitochondrial synthesis of ATP and insufficient energy provision. In the current study, we tested the efficiency of gene complementation by using a transgenic rescue approach in spontaneously hypertensive rats with the targeted *Tmem70* gene (SHR-*Tmem70^ko^*^/*ko*^), which leads to embryonic lethality. We generated SHR-*Tmem70^ko^*^/*ko*^ knockout rats expressing the *Tmem70* wild-type transgene (SHR-*Tmem70^ko^*^/*ko,tg*/*tg*^) under the control of the EF-1α universal promoter. Transgenic rescue resulted in viable animals that showed the variable expression of the *Tmem70* transgene across the range of tissues and only minor differences in terms of the growth parameters. The TMEM70 protein was restored to 16–49% of the controls in the liver and heart, which was sufficient for the full biochemical complementation of ATP synthase biogenesis as well as for mitochondrial energetic function in the liver. In the heart, we observed partial biochemical complementation, especially in SHR-*Tmem70^ko^*^/*ko,tg*/*0*^ hemizygotes. As a result, this led to a minor impairment in left ventricle function. Overall, the transgenic rescue of *Tmem70* in SHR-*Tmem70^ko^*^/*ko*^ knockout rats resulted in the efficient complementation of ATP synthase deficiency and thus in the successful genetic treatment of an otherwise fatal mitochondrial disorder.

## 1. Introduction

Mitochondrial diseases represent deleterious disorders of the mitochondrial oxidative phosphorylation apparatus (OXPHOS). Tissues with high energetic demands, such as brain, heart, and skeletal muscle tissues, are predominantly affected. Mitochondrial diseases are clinically presenting as encephalo-cardio-myopathies—severely devastating and often fatal conditions with an early manifestation, usually shortly after birth [1]. Given the dual genetic origin of OXPHOS complexes, their defects may arise both from mutations in mitochondrial (mtDNA) and nuclear (nDNA) genomes. Mutations in nuclear genes are the most typical for childhood-onset mitochondrial diseases, where they comprise 70–85% of identified cases [2,3].

Mitochondrial F_1_F_o_-ATP synthase (Complex V) is the final enzyme in the OXPHOS system that generates energy in form of ATP at the expense of proton motive force. The mammalian ATP synthase complex consists of 17 different subunits with total molecular weight of 650 kDa. Structurally, it is organised into two large domains: matrix-oriented hydrophilic F_1_ and membranous highly hydrophobic F_o_. These two major domains are attached to each other by the central and peripheral stalks. Subunits of the central stalk together with the oligomer of the c subunits (c-ring) form the rotor of the enzyme [4]. The biogenesis of the ATP synthase is rather complex process [5,6,7]. It proceeds via the assembly of several modules, starting with the independent formation of the F_1_ part and the c-ring. After the attachment of F_1_ to the membrane-embedded c-ring, the subunits of the peripheral stalk (b, d, F_6_ and OSCP) and of the membranous subcomplex (e, f, g, DAPIT and MLQ) are added. The final steps are represented by incorporation of the two mtDNA-encoded subunits (a and A6L), which are encoded by the *MT-ATP6* and *MT-ATP8* genes, respectively [8]. The formation of the ATP synthase from individual subunits was originally regarded as a stepwise process, but recent studies on a mammalian model demonstrated some level of variability regarding the incorporation of individual modules [9,10,11].

The biogenesis of the ATP synthase is assisted by numerous, enzyme-specific factors that partly differ between lower and higher eukaryotes. While several factors are involved in the assembly of mtDNA-encoded subunits in *S. cerevisiae* [5,12,13,14,15,16], none of them exist in mammals. Five assembly factors have been described for the mammalian enzyme. The first two, ATPAF1 (ATP11) and ATPAF2 (ATP12), are involved in the assembly of the α and β subunits into the α_3_β_3_ oligomer [5,6,7]. The third one—c7orf55 (FMC1) most likely represents a functional homologue of yeast Fmc1p, which interacts with ATP11 and ATP12 [17]. The fourth factor, TMEM70, is specific for higher eukaryotes [18]. It was originally characterised as the disease-causing gene [18,19,20,21], and we recently deciphered its molecular role in the biogenesis of F_o_-c subunit and in the assembly of the c8-ring in the enzyme rotor [22]. Most recently, an additional mitochondrial protein, TMEM242, was described as also being associated with F_o_-c subunit assembly [23]. Intriguingly, both TMEM70 and TMEM242 were also proposed as factors assisting Complex I (CI) biogenesis [23,24], which broadens the spectrum of potential phenotypic presentations.

ATP synthase represents key enzyme of mitochondrial energy provision responsible for the synthesis of as much as 90% of the ATP in human cells. ATP synthase disorders thus belong to the most severe metabolic diseases. They can be caused by mutations in either the mitochondrially or nuclearly encoded subunits of the enzyme. Regarding mitochondrial encoded subunits, the number of mutations in the *MT-ATP6* and/or *MT-ATP8* genes, which have been described to be associated with mitochondrial diseases, is quite high and steadily growing. The underlying mechanism in ATP synthase deficiency and thus in the clinical manifestation of these mutations may vary significantly from mutation to mutation [25,26]. The situation is further complicated by different levels of mutated mtDNA within cells and tissues of patients affected by these disorders. As for mutations in nuclear genes, up until now, five genes were associated with human mitochondrial diseases. Three of them, *ATP5A1* [27], *ATP5D* [28], and *ATP5E* [29] encode the enzyme’s structural α, δ, and ε subunits, respectively, while the other two, *ATPAF2* [30] and *TMEM70* [18], encode specific ancillary factors that facilitate the biogenesis of the ATP synthase. All of these defects share a similar biochemical phenotype with a pronounced decrease in the content of the fully assembled and functional ATP synthase complex. While mutations in the *ATP5A1*, *ATP5D*, *ATP5E,* and *ATPAF2* genes are very rare, *TMEM70* mutations represent the most frequent cause of ATP synthase deficiency, with over 20 different mutations reported in more than 50 affected families [26,31,32,33,34,35]. Apparently, the *TMEM70* gene is highly prone to mutagenesis, and this type of the rare mitochondrial disease has a rather frequent incidence.

While mitochondrial myopathies are devastating genetic diseases, there are almost no causal therapies available for patients [36,37]. In general, mitochondrial disorders may be approached by either pharmaceutic interventions, upregulating respective metabolic pathways, or by the means of classical gene therapy. However, despite the enormous progress that has recently been made in gene-editing approaches, genetic treatments for mitochondrial disorders are still unavailable [38].This is predominantly due to their extreme genetic and phenotypic variation, but complicated mitochondrial organisation plays a role as well. The mitochondrial biosynthetic apparatus is shielded by two mitochondrial membranes; thus, targeting genome editing tools towards mtDNA represents a major challenge. Therefore, mitopathies of nuclear origin may represent easier targets for gene therapy since they can share tools with other genetic treatments. At the preclinical level, successful gene therapy was achieved in several animal models of mitochondrial disorders. These include an *Ethe1^−^*^/*−*^ model of ethylmalonic encephalopathy (EE) [39], a *Tymp^−^*^/*−*^ model of mitochondrial neuro-gastrointestinal encephalo-myopathy (MNGIE) [40,41], and *Nr2f1^−^*^/*−*^ and *OPA1*^delTTAG^ models for autosomal dominant optic atrophy (ADOA, BBSOA) [42,43] as well as an *Ndufs4^−^*^/*−*^ model of Leigh syndrome [44,45].

Considering the unique properties of ATP synthase deficiency due to TMEM70 dysfunction, TMEM70 defects can be promising targets for establishing gene therapy. First, the absence of TMEM70 does not completely disrupt the biogenesis of the ATP synthase, and 20–30% of the assembled enzyme can be found in patients [18,46] as well as in an animal knockout model [21]. Second, the full recovery of ATP production can even be expected after only a partial increase in ATP synthase content, as this enzyme is present in effective excess in wild-type cells and tissues [47,48,49]. Third, the expression of wt*TMEM70* in patient fibroblasts lacking the TMEM70 protein restored ATP synthase content and function as well as mitochondrial ATP production [18,50].

In the present study, we attempted to complement a fatal *Tmem70* deficiency in a rat model. Laboratory rats have represented the model of choice for cardiovascular research for more than a century [51]. Its major advantage lies in their size and larger blood volume compared to mice as well as their better accessibility in contrast to larger animals [52]. The feasibility of rat model has dwindled since transgenic technologies were first pioneered in mice, but recent advances also make genetic manipulation available for rats [53,54,55,56]. We performed our studies in SHR (spontaneously hypertensive rat) inbred rats since they represent one of the best characterized rat models of cardiac hypertrophy [57], which is associated also with *Tmem70* dysfunction in humans [33]. There is also a plethora of available reference phenotypic data for SHR rats, which makes it ideal for research involving metabolic phenotypes. Ultimately, we have good previous experience with transgenic technology in this strain [53,54,55,56].

We based our studies on a recently established SHR *Tmem70* knockout line (SHR-*Tmem70^ko^*^/*ko*^). Similar to our previously developed mouse model, homozygous SHR-*Tmem70^ko^*^/*ko*^ results in embryonic lethality [21]. Therefore, we induced the transgenic expression of the *Tmem70* wild-type gene using the Sleeping Beauty transposon system to characterize the organismal and organ-specific response to *Tmem70* transgenic expression under the control of the EF-1α universal promotor. The transgenic restoration of TMEM70 protein content reached 16–49% of that in the controls in the liver and heart, which was sufficient for full biochemical complementation (ATP synthase biogenesis and content, mitochondrial energetic function) in the liver. In the heart, we observed partial biochemical complementation, especially in SHR-*Tmem7^ko^*^/*ko,Tg*/*0*^ hemizygotes. As a result, this led to the minor impairment of the functional parameters in the heart.

Overall, EF-1α-driven *Tmem70* transgenesis in SHR-*Tmem70^ko^*^/*ko*^ knockout rats resulted in an efficient complementation of the deficiency in the ATP synthase and thus enabled the successful genetic treatment of an otherwise fatal mitochondrial disorder.

## 2. Materials and Methods

### 2.1. Animals

The SHR/OlaIpcv (referred to as SHR), SHR-*Tmem70^tm^*^1^/OlaIpcv (tm1 – targeted mutation 1) knockout (referred to as SHR-*Tmem70^ko^*^/*ko*^), SHR-Tg(Ef1a-*Tmem70*)OlaIpcv transgenic (referred to as SHR-*Tmem70^tg^*^/*tg*^), and SHR-*Tmem70*^tm1^-Tg(Ef1a-*Tmem70*)OlaIpcv (referred to as SHR-*Tmem70^ko^*^/*ko,tg*/*tg*^) transgenically rescued rats used in the current study were housed in an air-conditioned animal facility and were allowed free access to standard laboratory chow and water. Sample collection was performed from animals in a postprandial state under 2% isoflurane anaesthesia. Both biochemical and echocardiography phenotypes were analysed in 14-week-old male rats.

### 2.2. Generation of Tmem70 Knockout Rats

*Tmem70* knockout rats were generated by microinjecting fertilized SHR ova with the ZFN (Zinc Finger Nuclease) construct (Merck KGaA, Darmstadt, Germany), as described elsewhere [58]. In brief, the ZFN construct was designed to target the first exon of the *Tmem70* gene. Altogether, three positive animals were detected through the screening of the PCR products using the Surveyor mutation detection kit (Integrated DNA Technologies, Inc., Coralville, Iowa 52241, USA). An SHR-*Tmem70*^tm1^/Ipcv line no. 154 (referred to as SHR-*Tmem70^ko^*^/*ko*^) with a 131 bp deletion (nt 236–366) was selected for further analyses. Homozygous mutants are embryonically lethal at ≈E9, but heterozygous survival was achieved without major developmental abnormalities [58].

### 2.3. Generation of Tmem70 Transgene

Three independent SHR-*Tmem70^tg^*^/*tg*^ transgenic lines (lines 116, 126 and 130) were derived by microinjecting SHR fertilized ova with a mix of Sleeping Beauty construct encoding wild-type (WKY) *Tmem70* cDNA under the control of the universal EF-1α promoter and mRNA from the SB100X transposase, as previously described [59].

### 2.4. Genotyping

DNA from rat tissues was isolated using Invisorb Spin Tissue Mini Kit (Stratec Biomedical Systems, Birkenfeld, Germany). PCR was run with AmpliTaq Gold 360 polymerase (Thermo Fisher Scientific, Waltham, MA 02451, USA), 0.2 mM dNTPs (Merck KGaA, Darmstadt, Germany), and 0.5 μM of the following primers (Generi Biotech s.r.o., Hradec Králové, Czech Republic): TMEM70 ZFN-F CAGCATGTTGTTGCCTATGG, TMEM70 ZFN-R AGACCACACAATCTGGGACC. PCR products were resolved by 1.5% agarose gel electrophoresis. The presence of the *Tmem70* transgene was evaluated by qPCR using primers, allowing amplification specifically from vector *Tmem70* cDNA containing no introns. Primers were placed in exon 1 (F- GGCAGGCTGATTTATACTGGGA) and exon 2 (R- AAGGCTGACCACACTTGTAGA). The albumin gene was used as a control (F- AAGACGGCCATGTTTCTCTG, R- TGGAAGGTGAAGGTCTCAGC). Amplification was carried out with PowerUp SYBR Green Master Mix (Thermo Fisher Scientific, Waltham, MA 02451, USA) at standard reaction conditions on the Viia7 instrument (Thermo Fisher Scientific, Waltham, MA 02451, USA). All reactions were conducted in triplicate. The allelic content of the *Tmem70* transgene was calculated by the 2^−ΔΔCt^ relative quantification method using Quant Studio software (Thermo Fisher Scientific, Waltham, MA 02451, USA).

### 2.5. Gene Expression Analysis

The total RNA was isolated from the tissues using the RNeasy Plus Universal Mini Kit (Qiagen, Germany), and cDNA was synthesized from 0.5 μg of RNA by reverse transcription (SCRIPT cDNA Synthesis Kit, Jena Bioscience, Jena, Germany). The following primer sets were used: total *Tmem70* (F- CCTGGCCCGGACAGTATTTG, R- CGGTAAGAATGCAAGGCTGAC), transgenic *Tmem70* (F- TCGGTCTCGATTCTACGCGA, R- GCAAACAACAGATGGCTGGC), and *Hprt* (F- ACCAGTCAACGGGGGACATA, R- TTGGGGCTGTACTGCTTGAC). qPCR amplifications were carried out on a ViiA 7 instrument (Thermo Fisher Scientific, Waltham, MA 02451, USA) with the following cycling protocols: 95 °C for 12 min and 40 cycles at 95 °C 15 s, 60 °C for 20 s, and 72 °C for 20 s. All of the reactions were conducted in triplicate, and 1.5 µL of diluted (1:10) cDNA was used in each 5 µL reaction using HOT FIREPol EvaGreen qPCR Mix Plus (Solis Biodyne, Tartu, Estonia). Transcript quantity was calculated in Quant Studio SW (Thermo Fisher Scientific, Waltham, MA 02451, USA) based on standard curves for individual genes created by serially diluting a 1:1 mixture of wild-type and transgenic liver cDNA. *Hprt* transcript levels were used as a housekeeper reference.

### 2.6. Tissue Homogenates

Tissue homogenates (7.5–10%, *w*/*v*) were prepared at 4 °C in STE medium (0.25 (for heart) or in 0.35 (for liver) M sucrose, 10 mM Tris-HCl, 2 mM EDTA, pH 7.4 containing protease inhibitor cocktail, PIC 1:1000, P8340, Merck KGaA, Darmstadt, Germany) using glass-Teflon homogenizer and filtered through a fine mesh [60]. Heart samples were then re-homogenised with Dounce glass-glass homogeniser. All of the homogenates were used fresh for respirometric analyses or were stored at −80 °C for other assays. For SDS-PAGE, frozen tissue samples were milled with zirconium oxide grinding balls (3 min 30 Hz) using a Retsch MM 400 mixer mill (Retsch, Haan, Germany) in the same buffers as those mentioned above. Protein content was measured using the Bradford method [61].

### 2.7. Electrophoretic Analyses

Samples for SDS-PAGE were denatured for 30 min at 37 °C in a sample lysis buffer containing 50 mM Tris-HCl pH 7.0, 4% (*w*/*v*) SDS, 10% (*v*/*v*) glycerol, 0.02% (*w*/*v*) Coomassie Brilliant Blue R-250, and 2% (*v*/*v*) 2-mercaptoethanol and were separated on 12% polyacrylamide minigels using the Tricine buffer system [62].

For native electrophoresis, tissue homogenates were centrifuged for 15 min at 20,000× *g* at 4 °C to obtain a membrane fraction. These pellets were resuspended in buffer containing 50 mM NaCl, 2 mM 6-aminohexanoic acid, 50 mM imidazole, and 1 mM EDTA with pH 7, solubilized with digitonin (4 g/g protein) for 20 min on ice, and centrifuged for 20 min at 30,000× *g*. To the resulting supernatants, 5% glycerol and Coomassie Brilliant Blue G-250 (dye/detergent ratio 1:8) were added, and the samples were subsequently analysed by means of blue native electrophoresis (BNE) using 4–13% polyacrylamide gradient mini gels and the imidazole buffer system [63].

### 2.8. In-Gel Activity Staining

The ATPase hydrolytic activity assay [64] was used to visualize active ATP synthase complexes in the native gels. Subsequently, the gels were washed in distilled water and were scanned on a flatbed scanner. The scanned images were quantified by ImageLab 6.0 software (Bio-Rad Laboratories, Inc., Hercules, CA 94547, USA).

### 2.9. Western Blot Analysis

Gels were blotted onto a PVDF (polyvinylidene difluoride) membrane (Immobilon FL 0.45 μm, Merck KGaA, Darmstadt, Germany) by means of semi-dry electro-transfer (1 h at 0.8 mA/cm^2^) using a Transblot SD apparatus (Bio-Rad Laboratories, Inc., Hercules, CA 94547, USA). The PVDF membranes were washed for 5 min in TBS (150 mM Tris- HCl, 10 mM NaCl; pH 7.5) and were blocked in 5% (*w*/*v*) fat-free dry milk diluted in TBS for 1 h. Then, the membranes were washed 3 × 10 min in TBST (TBS with 0.1% (*v*/*v*) detergent Tween-20), as previously described [65]. For Western blot (WB) immunodetection, the membranes were incubated in primary antibody (2 h at room temperature or overnight at 4 °C). We used antibodies to subunits of the ATP synthase (F_1_-α [66] lot 20D6, F_1_-β (Abcam, Cambridge, United Kingdom, ab14730), F_o_-a (refs 20), F_o_-b (Abcam ab117991), F_o_-c (Abcam ab180149), Complex I (NDUFA9 (Abcam ab14713), NDUFS3 (Abcam ab110246), NDUFB8 (Abcam ab110242)), Complex II (SDHA, Abcam ab14715), Complex III (Core 2, Abcam ab14745), Complex IV (COX4, Abcam ab110261), and TMEM70 (Santa Cruz Biotechnology, Dallas, TX 75220, USA, sc-135004). For quantitative detection, the corresponding infra-red fluorescent secondary antibodies (Alexa Fluor 680, Thermo Fisher Scientific, Waltham, MA 02451, USA; IRDye 800, LI- COR Biosciences, Lincoln, NE, USA, 68504-5000) were used. Detection was performed using the Odyssey fluorescence scanner (LI-COR Biosciences, Lincoln, NE, USA, 68504-5000), and signals were quantified by ImageLab 6.0 software (Bio-Rad Laboratories, Inc., Hercules, California 94547, USA).

### 2.10. High Resolution Oxygraphy

Oxygen consumption was measured at 30 °C using the Oxygraph-2k (Oroboros Instruments GmbH, Innsbruck, Austria) as previously described [67]. The homogenate was suspended (0.05–0.15 mg/mL) in mannitol-based medium (70 mM sucrose, 220 mM mannitol, 10 mM KH_2_PO_4_, 5 mM MgCl_2_, 2 mM HEPES, 1 mM EGTA, 0.2 mg/mL BSA, pH 7.4), and for the measurements, respiratory substrates and inhibitors were used at following concentrations: 50 μM palmitoyl carnitine, 2 mM malate, 10 mM pyruvate, 10 mM glutamate, 10 mM succinate, 5 μM cytochrome *c*, 1 mM ADP, 5–100 nM oligomycin, 1–3 μM FCCP, 1 μM rotenone, and 10 mM malonate. The oxygen consumption rates were analysed using DatLab 5 software (Oroboros Instruments GmbH, Innsbruck, Austria) and were expressed in pmol O_2_/s/mg protein. The respiratory control index (RCI) was calculated as a ratio between the fully substrate-saturated coupled rate and leak respiration induced by the addition of oligomycin.

### 2.11. Echocardiography

In the separate sets of rats, the evaluation of the geometrical and functional parameters of the hearts was performed using the Vivid 7 (GE Healthcare, Chicago, IL, USA) echocardiographic system with a 14 MHz linear matrix probe [68]. The diastolic and systolic dimensions of the left ventricle (LV) were measured during echocardiography evaluation, as were the anterior and posterior wall thickness (AWTd, PWTd, AWTs, PWTs) and the LV cavity diameter (LVDd, LVDs). From these dimensions, the following functional echocardiography parameters were derived using the formulas: fractional shortening (FS) = 100 ∗ (LVDd − LVDs)/LVDd, ejection fraction (EF) = 100 ∗ (LVDd^3^ − LVDs^3^)/LVDd^3^.

## 3. Results

### 3.1. Generation of Tmem70 Knockout SHR Expressing Tmem70 Wild-Type Transgene (SHR-Tmem70^ko/ko,tg/tg^)

We previously established a mouse model for *Tmem70* knockout [21], which showed embryonic lethality at embryonal day 9.5. To achieve greater phenotype variability, we attempted to produce a *Tmem70* dysfunction model in rats. For this purpose, we used Zinc Finger Nuclease mutagenesis (Sigma) in an SHR (spontaneously hypertensive rat) strain.

Using this approach, we generated several mutant lines, and for further experiments, we used SHR-*Tmem70*^tm1^/Ipcv line no. 154 (referred to as SHR-*Tmem70^ko^*^/*ko*^), which harbours 131 bp deletion (nt 236–366) that disrupts the exon 1 boundary and leads to the total absence of the TMEM70 protein. Similar to *Tmem70* knockout mice [21], homozygous SHR-*Tmem70^ko^*^/*ko*^ rats were embryonically lethal at ≈E9, but the SHR-*Tmem70^ko^*^/*wt*^ heterozygotes are alive and were able to survive without experiencing any major developmental abnormalities.

**Figure 1:**
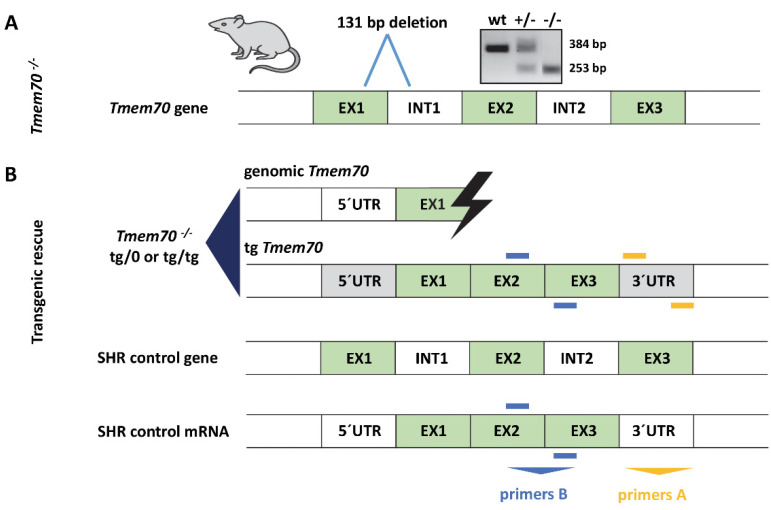
Spontaneously hypertensive rat (SHR)-*Tmem70*^ko/ko^ production and *Tmem70* transgenic complementation. (**A**) Generation of SHR-*Tmem70^ko^*^/*ko*^ rats by ZFN-induced deletion of 131 bp in exon 1. (**B**) Transgenic rescue by crossing of SHR-*Tmem70^ko^*^/*wt*^ knockout heterozygotes with SHR-*Tmem70^tg^*^/*tg*^ transgenic rats expressing wild-type *Tmem70* transgene under control of the universal EF-1α promoter. Orange bars (primers A) indicate the position of primers that are specific for transgene construct; blue bars (primers B) represent the position of the primers used for the quantification of the total *Tmem70* transcript—endogenous and transgenic. EX—exon, INT—intron, UTR—untranslated region.

A lack of a viable homozygous SHR-*Tmem70^ko^*^/*ko*^ offspring prevents the postnatal testing of any AAV (adeno-associated virus) mediated complementation. Therefore, as a proof of concept, we turned to the transgenic expression of wild-type *Tmem70* using the Sleeping Beauty (SB) transposon system on the genetic background of SHR-*Tmem70^ko^*^/*ko*^. To achieve this objective, we first derived SHR-*Tmem70^tg^*^/*tg*^ transgenic animals and crossed them with SHR-*Tmem70^ko^*^/*wt*^ heterozygotes (Figure 1). Three lines of SHR rats with a homozygous expression of the *Tmem70* transgene (SHR-*Tmem70^tg^*^/*tg*^) were genetically fixed (lines 126, 130 and 116), and we produced *Tmem70* knockout rats expressing *Tmem70* transgene thorough subsequent intercrosses. Animals that were either homozygous (SHR-*Tmem70^ko^*^/*ko,tg*/*tg*^) or hemizygous (SHR-*Tmem70^ko^*^/*ko,tg*/*0*^) for the transgene were used for further analyses.

### 3.2. Tmem70 Transgene Expression in SHR Rats

**Figure 2:**
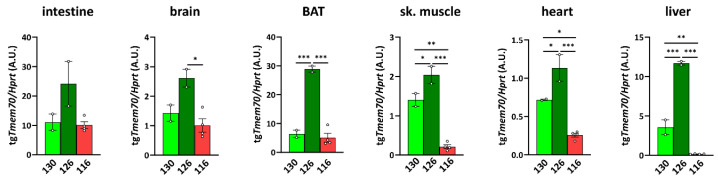
*Tmem70* transgene expression in SHR rats across tissues and lines. Transgenic *Tmem70* mRNA levels were analysed in the intestine, brain, brown adipose tissue (BAT), skeletal muscle, heart, and liver of three animal lines, 130, 126, and 116, and normalized to *Hprt* expression. Data are mean ± SEM, *n* = 2–4, * *p* ≤ 0.05, ** *p* ≤ 0.01, *** *p* ≤ 0.001.

To characterize the expression of the *Tmem70* transgene that is driven by the EF-1α universal promotor, we analysed *Tmem70* transgene transcripts by means of quantitative RT-PCR in the animal tissues from the three SHR-*Tmem70^tg^*^/*tg*^ lines. For this purpose, we utilised primer pair A (Figure 1), which takes advantage of the different 3′ UTR sequence of the transgene construct. Transgene expression of *Tmem70* showed difference of up to 10-fold in the tested tissues, with the highest levels of the *Tmem70* transgene transcripts being detected in the intestine and liver and the lowest levels being detected in the heart (brown adipose tissue (BAT) ≥ intestine > liver > brain ≥ skeletal muscle > heart) (Figure 2).

Clearly, the highest level of transgenic expression was achieved in line 126 since *Tmem70* transgene expression was significantly higher in this line when compared to the other two lines in all of the tissues except for in the intestine, where higher expression trends were apparent. Lines 130 and 116 displayed similar levels of *Tmem70* transgene expression in the intestine, brain, and BAT, but in line 116, the detected *Tmem70* transgene expression was significantly lower in the skeletal muscle, heart, and liver. Interestingly, in the subsequent crossing with the SHR-*Tmem70^ko^*^/*wt*^ rats, only the animals from lines 126 and 130 produced viable offspring of the SHR-*Tmem70^ko^*^/*ko,tg*/*0*^ genotype, indicating that the transgene expression levels in heart, muscle, and liver of line 116 were too low, meaning that this was probably responsible for unsuccessful complementation.

### 3.3. Impact of Tmem70 Transgene Expression on Growth Phenotype

**Figure 3:**
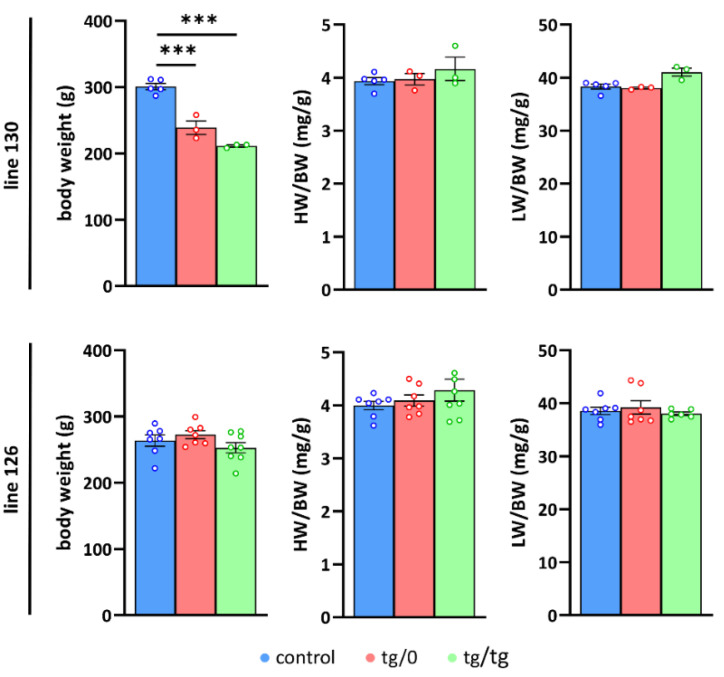
Growth parameters in *Tmem70* transgenic rats. Body weight (BW) and heart weight (HW) or liver weight (LW) relative to body weight (LW/BW, HW/BW) were analysed in control SHR rats and in SHR-*Tmem70^ko^*^/*ko,tg*/*0*^ (tg/0) and SHR-*Tmem70^ko^*^/*ko,tg*/*tg*^ (tg/tg) rats derived from transgenic lines 130 and 126). Data are mean ± SEM, *n* = 3–8, *** *p* ≤ 0.001.

In subsequent experiments, we only studied animals from the successfully complemented lines: 126 and 130, and followed their phenotypes in either the homozygotes (SHR-*Tmem70^ko^*^/*ko,tg*/*tg*^) or hemizygotes (SHR-*Tmem70^ko^*^/*ko,tg*/*0*^) for the *Tmem70* transgene. To evaluate the growth parameters of the transgene-complemented SHR-*Tmem70^ko^*^/*ko*^ animals in lines 130 and 126, we compared them to the control SHR rats (Figure 3). We found that the rats from line 130 had a reduced body weight of 79 ± 3% for the SHR-*Tmem70^ko^*^/*ko,tg*/*0*^ rats and of 70 ± 1% for the SHR-*Tmem70^ko^*^/*ko,tg*/*tg*^ animals relative to the SHR controls.

No significant difference was found between the SHR-*Tmem70^ko^*^/*ko,tg*/*tg*^ and SHR-*Tmem70^ko^*^/*ko,tg*/*0*^ animals, indicating that the transgenic complementation for both genotypes from the line 130 had similar efficiency. We did not find any signs of either heart or liver hypertrophy (heart or liver weight expressed relative to body weight) in line 130. Concordantly, the analysis of line 126 did not show any decrease in either parameter compared to controls, likewise suggesting full complementation in both the SHR-*Tmem70^ko^*^/*ko,tg*/*tg*^ and SHR-*Tmem70^ko^*^/*ko,tg*/*0*^ rats. Interestingly, growth retardation was absent in the line 126, which may be a consequence of better complementation due to higher transgene expression.

### 3.4. Complementation of ATP Synthase Deficiency

**Figure 4:**
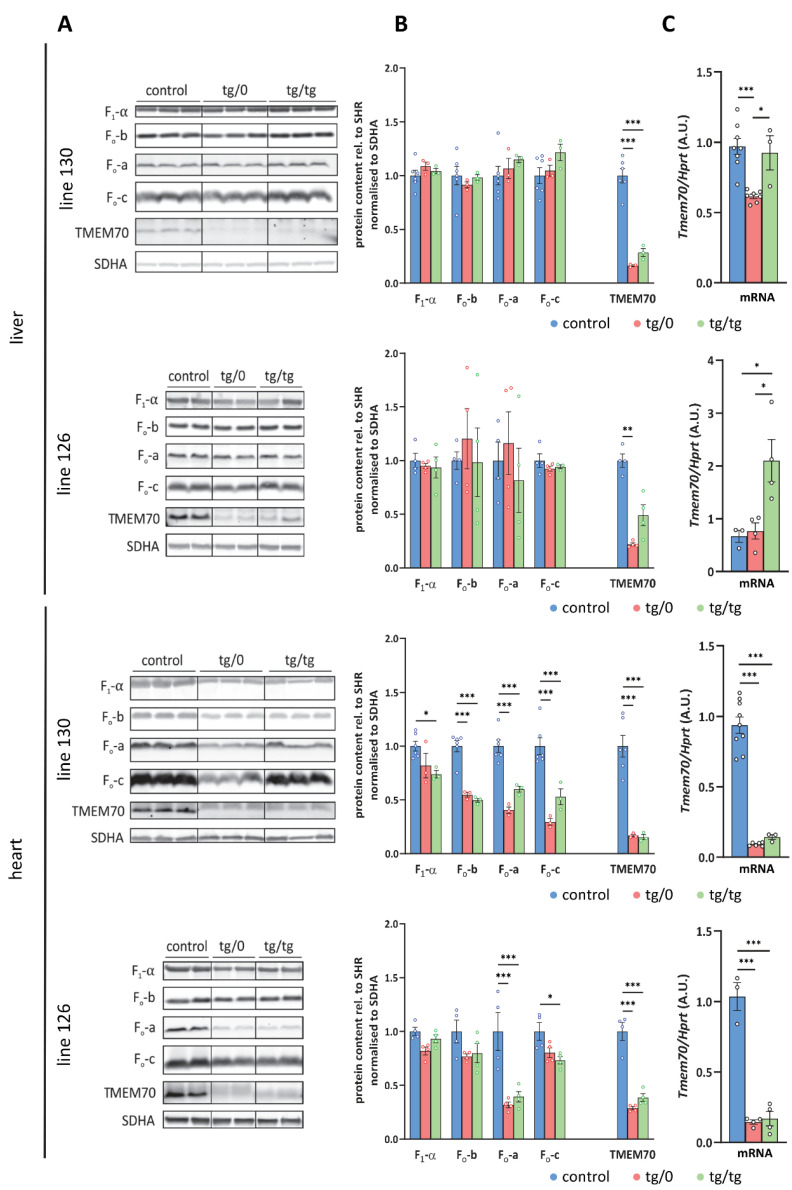
Transgenic complementation of ATP synthase deficiency. (**A**) WB detection of ATP syn-thase subunits in liver and heart homogenates and (**B**) quantification of the content of the F1-α, Fo-a, Fo-b, and Fo-c subunits relative to the SDH content (SDHA) in the control SHR rats and in the SHR-Tmem70ko/ko,tg/0 (tg/0) or SHR-Tmem70ko/ko,tg/tg (tg/tg) rats derived from SHR-Tmem70tg/tg trans-genic lines 130 and 126. (**C**) Tissue Tmem70 mRNA levels were analysed by RT-PCR and normalized to HPRT mRNA levels. Data are mean ± SEM, *n* = 3–6, * *p* ≤ 0.05, *** *p* ≤ 0.001.

To examine how *Tmem70* transgenic rescue affects ATP synthase deficiency associated with ATP synthase dysfunction, we initially analysed the protein levels of representative structural subunits of the ATP synthase as well as the levels of the TMEM70 protein itself (Figure 4). We focused on the liver and heart; these tissues were primarily affected in patients with TMEM70 dysfunction [18,33] and, as apparent from *Tmem70* transgene expression requirements (Figure 2), were also crucial for the successful complementation of *Tmem70* knockout in the rat model.

An analysis of liver homogenates (Figure 4A,B) revealed the control levels for all of the assessed ATP synthase subunits, namely F_1_-α (F_1_ catalytic domain), F_o_-b (peripheral stalk) and F_o_-a and F_o_-c (key subunits from F_o_ domain) in either the SHR-*Tmem70^ko^*^/*ko,tg*/*tg*^ or SHR-*Tmem70^ko^*^/*ko,tg*/*0*^ animals from both the 130 and 126 lines. This points towards complete ATP synthase restoration in liver, which was achieved just by the transgenic expression of *Tmem70* from a single allele (SHR-*Tmem70^ko^*^/*ko,tg*/*0*^) alone. The ATP synthase subunits reached their control levels despite only partial restoration of the TMEM70 protein content being achieved. The detected transgenic TMEM70 protein levels were 16.3 ± 0.6% for the SHR-*Tmem70^ko^*^/*ko,tg*/*0*^ animals and 28.5 ± 2.6% for the SHR-*Tmem70^ko^*^/*ko,tg*/*tg*^ animals from line 130 and 22 ± 2.8% for the SHR-*Tmem70^ko^*^/*ko,tg*/*0*^ animals and 49 ± 19.5% for the SHR-*Tmem70^ko^*^/*ko,tg*/*tg*^ animals in case of strain 126, indicating the gene dosage effect. This suggests that even minuscule levels of the TMEM70 protein are sufficient for the full complementation of ATP synthase deficiency, including the content of subunit c — the target client for TMEM70 [22]. 

**Figure 5:**
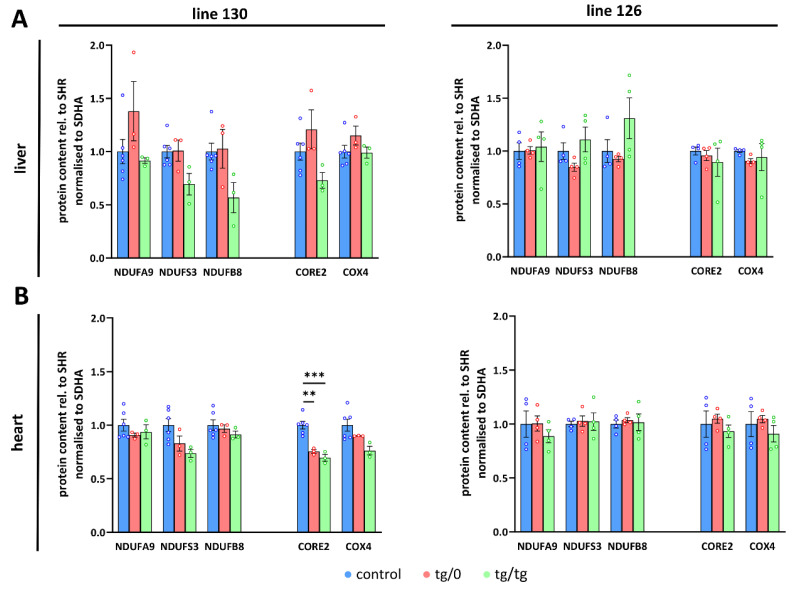
Respiratory chain complexes in complementation of ATP synthase deficiency. WB quantification in (**A**) liver and (**B**) heart of subunits of the Complex I (CI, NDUFA9, NDUFS3, NDUFB8), III (CIII, CORE2), and IV (CIV, COX4) related to Complex II (CII, SDHA) content was performed in control SHR rats and in SHR-*Tmem70^ko^*^/*ko,tg*/*0*^ (tg/0) or SHR-*Tmem70^ko^*^/*ko,tg*/*tg*^ (tg/tg) rats derived from SHR-*Tmem70^tg^*^/*tg*^ transgenic lines 130 and 126. Data are mean ± SEM, *n* = 3–6, ** *p* ≤ 0.01, *** *p* ≤ 0.001.

Additionally, we also determined *Tmem70* mRNA expression. For this purpose, we used primer pair B (Figure 1), which quantifies both wild-type and transgenic *Tmem70* and allows for a comparison between native and transgenic expression. In line 130, the transcripts in the SHR-*Tmem70^ko^*^/*ko,tg*/*0*^ animals were significantly lower than they were in the controls (63.3 ± 1.6%) and were fully complemented in SHR-*Tmem70^ko^*^/*ko,tg*/*tg*^ rats (95.3 ± 12.5%). To the contrary, in line 126, even one allele in the SHR-*Tmem70^ko^*^/*ko,tg*/*0*^ animals led to the restoration of the *Tmem70* transcripts (89.9 ± 13.5% of control), and in the SHR-*Tmem70^ko^*^/*ko,tg*/*tg*^ animals, they were even increased to 268.6 ± 82.6% compared to in the controls (Figure 4C). To assess the possible effect on other respiratory chain complexes, we also quantified the steady state content of the representative subunits from Complex I (NADH dehydrogenase—subunits NDUFA9, NDUFS3, and NDUFB8), Complex III (CIII, coenzyme Q: cytochrome *c* oxidoreductase—subunit CORE2), and Complex IV (CIV, cytochrome *c* oxidase—subunit COX4). No significant difference were observed in either case; however, there was a tendency towards a decrease in the content of the CI subunits in the case of the SHR-*Tmem70^ko^*^/*ko,tg*/*tg*^ animals that had been derived from line 130 (Figure 5A).

A different picture emerged from the analysis of the heart homogenates. As shown in Figure 4A,B, only partial complementation of the ATP synthase subunits levels was achieved. For the SHR-*Tmem70^ko^*^/*ko,tg*/*0*^ and SHR-*Tmem70^ko^*^/*ko,tg*/*tg*^ animals derived from line 130, the decrease was significant in all cases except for F_1_-α in the SHR-*Tmem70^ko^*^/*ko,tg*/*0*^ animals. The best complementation can be seen for F_1_-α, while the content of the F_o_ subunits b, a, and c remains more profoundly reduced, pointing towards the less pronounced restoration of the F_o_ membrane sector subunits by *Tmem70* transgenesis. The complementation was more efficient in SHR-*Tmem70^ko^*^/*ko,tg*/*tg*^ compared to in SHR-*Tmem70^ko^*^/*ko,tg*/*0*^ in the case of F_o_-a and especially in the case of the F_o_-c subunit. In accordance with previous results, the SHR-*Tmem70^ko^*^/*ko,tg*/*0*^ and SHR-*Tmem70^ko^*^/*ko,tg*/*tg*^ animals that had been derived from line 126 displayed better complementation overall, yet the pattern stayed similar to the pattern seen in line 130. Thus, only the contents of subunits F_o_-a and F_o_-c were significantly reduced in both the SHR-*Tmem70^ko^*^/*ko,tg*/*0*^ and SHR-*Tmem70^ko^*^/*ko,tg*/*tg*^ animals. In the hearts of both the SHR-*Tmem70^ko^*^/*ko,tg*/*0*^ and SHR-*Tmem70^ko^*^/*ko,tg*/*tg*^ animals that had been derived from lines 130 and 126, only partial restoration of the TMEM70 protein content was achieved, and it was even lower than the restoration in the liver. However, it was higher in the SHR-*Tmem70^ko^*^/*ko,tg*/*0*^ and SHR-*Tmem70^ko^*^/*ko,tg*/*tg*^ animals that had been derived from line 126 (28.6 ± 2.9% for SHR-*Tmem70^ko^*^/*ko,tg*/*0*^, 38.6 ± 7% for SHR-*Tmem70^ko^*^/*ko,tg*/*tg*^) than it was in the SHR-*Tmem70^ko^*^/*ko,tg*/*0*^ and SHR-*Tmem70^ko^*^/*ko,tg*/*tg*^ animals that had been derived from line 130 (16.7 ± 0.9% for SHR-*Tmem70^ko^*^/*ko,tg*/*0*^, 15.3 ± 1.6% for SHR-*Tmem70^ko^*^/*ko,tg*/*tg*^). The same expression profile was obtained via the analysis of the *Tmem70* mRNA levels (Figure 4C), thus confirming that biochemical complementation is primarily highly dependent on *Tmem70* transgene expression. The content of all of the heart respiratory chain complexes (Figure 5B) was identical in the SHR-*Tmem70^ko^*^/*ko,tg*/*0*^ and SHR-*Tmem70^ko^*^/*ko,tg*/*tg*^ animals that had been derived from transgenic line 126, while in the animals that had been derived from line 130, the CIII content was slightly decreased in both the SHR-*Tmem70^ko^*^/*ko,tg*/*0*^ and SHR-*Tmem70^ko^*^/*ko,tg*/*tg*^ rats.

### 3.5. Mitochondrial Energetic Function

**Figure 6:**
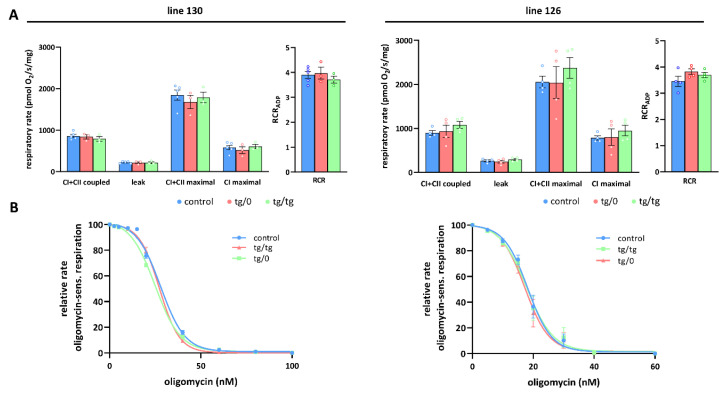
Full complementation of respiratory chain function in the liver. (**A**) Oxygen consumption in tissue homogenates was measured with CI (palmitoyl carnitine+pyruvate+glutamate) and CII (succinate) substrates in the presence of ADP (CI+CII coupled), oligomycin (leak), and FCCP (CI+CII maximal or CI maximal), RCR—respiratory control ratio. (**B**) Titration of sensitivity to oligomycin of ADP-stimulated respiration (CI+CII coupled) was analysed in control SHR rats and in SHR-*Tmem70^ko^*^/*ko,tg*/*0*^ (tg/0) or SHR-*Tmem70^ko^*^/*ko,tg*/*tg*^ (tg/tg) derived from SHR-*Tmem70^tg^*^/*tg*^ transgenic lines 130 and 126. Data are mean ± SEM, *n* = 3–5.

**Figure 7:**
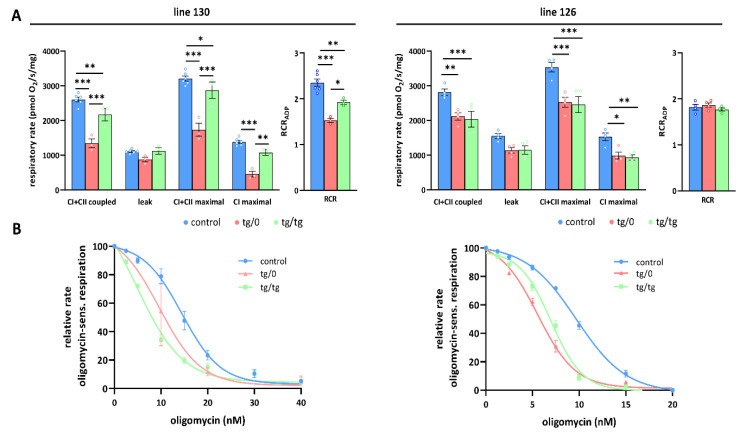
Partial complementation of respiratory chain function in heart. (**A**) Oxygen consumption in tissue homogenates was measured with CI (palmitoyl carnitine+pyruvate+glutamate) and CII (succinate) substrates in the presence of ADP (CI+CII coupled), oligomycin (leak), and FCCP (CI+CII maximal or CI maximal), RCR—respiratory control ratio. (**B**) Titration of sensitivity to oligomycin of ADP-stimulated respiration (CI+CII coupled) was analysed in control SHR rats and in SHR-*Tmem70^ko^*^/*ko,tg*/*0*^ (tg/0) or SHR-*Tmem70^ko^*^/*ko,tg*/*tg*^ (tg/tg) derived from SHR-*Tmem70^tg^*^/*tg*^ transgenic lines 130 and 126. Data are mean ± SEM, *n* = 3–6 * *p* ≤ 0.05, ** *p* ≤ 0.01, *** *p* ≤ 0.001.

To further investigate how *Tmem70* transgene expression affected mitochondrial oxidative phosphorylation activity, we analysed oxygen consumption by the mitochondrial respiratory chain using high-resolution oxygraphy (Figure 6 and Figure 7). Respiratory measurements in tissue homogenates were performed using parallel substrate inputs for both CI (palmitoyl carnitine, pyruvate, glutamate) and CII (succinate), i.e., conditions that generate the highest levels of mitochondrial proton gradient (ΔµH^+^) and that allow for the best evaluation of the ADP-phosphorylating capacity of the ATP synthase. Oxygen consumption was measured in the presence of ADP (coupled) with a mixture of CI and CII substrates after inhibition with oligomycin (leak) and in the uncoupled state with FCCP (maximal). In the liver, we revealed no differences between both the SHR-*Tmem70^ko^*^/*ko,tg*/*0*^ and SHR-*Tmem70^ko^*^/*ko,tg*/*tg*^ animals that had been derived from lines 130 and 126 and from the control rats, pointing to the fact that we achieved full transgenic complementation of OXPHOS energetic function that was not limited by ATP synthase. Accordingly, the respiratory control ratio (RCR), the general measure of the energetic function of mitochondria, was the same in the transgenic and control rats (Figure 6A). The unaltered function of the ATP synthase was also illustrated by titration of State 3-ADP (coupled) respiration with oligomycin (Figure 6B), which showed no differences in inhibitor sensitivity between SHR-*Tmem70^ko^*^/*ko,tg*/*0*^ and SHR-*Tmem70^ko^*^/*ko,tg*/*tg*^ animals and in the SHR controls.

OXPHOS function measurements in the hearts from the SHR-*Tmem70^ko^*^/*ko,tg*/*0*^ and SHR-*Tmem70^ko^*^/*ko,tg*/*tg*^ animals derived from line 130 (Figure 7A) revealed significantly decreased rates of ADP-stimulated respiration (coupled) compared to the controls, and the rate was lower in SHR-*Tmem70^ko^*^/*ko,tg*/*0*^ (51.6 ± 4.9% of controls) than it was in the SHR-*Tmem70^ko^*^/*ko,tg*/*tg*^ rats (83.3 ± 7% of controls). Transgenically rescued rats also showed similarly decreased rates of state 3-uncoupled (maximal) respiration in the SHR-*Tmem70^ko^*^/*ko,tg*/*0*^ rats, while there were no differences between the transgenes and controls in terms of oligomycin-inhibited oxygen consumption (leak). The RCR followed the same pattern, indicating that decreased coupled respiration and lower ATP production in transgenic rats were due to limited ATP synthase capacity. This was also illustrated by the oligomycin titration (Figure 7B) of ADP-stimulated respiration, demonstrating higher sensitivity in SHR-*Tmem70^ko^*^/*ko,tg*/*0*^ and SHR-*Tmem70^ko^*^/*ko,tg*/*tg*^ rats compared to in the SHR controls. These functional data are in good agreement with the observed decrease in the ATP synthase content (Figure 4) and demonstrate that the incomplete restoration of the ATP synthase content has implications for OXPHOS function.

A similar yet much less pronounced decrease in coupled respiration not affecting the RCR was found in the SHR-*Tmem70^ko^*^/*ko,tg*/*0*^ and SHR-*Tmem70^ko^*^/*ko,tg*/*tg*^ animals derived from line 126 (Figure 7A). Hence, the transgenic complementation in the heart did not lead to the full recovery of mitochondrial energetic function due to insufficient restoration of the ATP synthase content and thus ADP-phosphorylating activity. Interestingly, in the hearts of both the SHR-*Tmem70^ko^*^/*ko,tg*/*0*^ and SHR-*Tmem70^ko^*^/*ko,tg*/*tg*^ animals derived from the 130 and 126 strains, we noted a significant reduction in the rate of uncoupled respiration both when supplying the respiratory chain with a combination of CI+CII substrates (CI+CII maximal) or with CI-linked (CI maximal) substrates alone (Figure 7A). Since this respiratory state is independent of the capacity of the ATP synthase, this also implicates that there is a possible dysfunction at the level of the respiratory chain complexes, possibly in CI.

### 3.6. Cardiac Function

In animals who achieved the lowest level of biochemical complementation in the heart (i.e., SHR-*Tmem70^ko^*^/*ko,tg*/*0*^ animals derived from line 130), we also focused on the evaluation of left ventricle (LV) geometry and function in vivo by echocardiography to see whether hearts with an observed mitochondrial energetic defect could sustain normal contractility.

**Figure 8:**
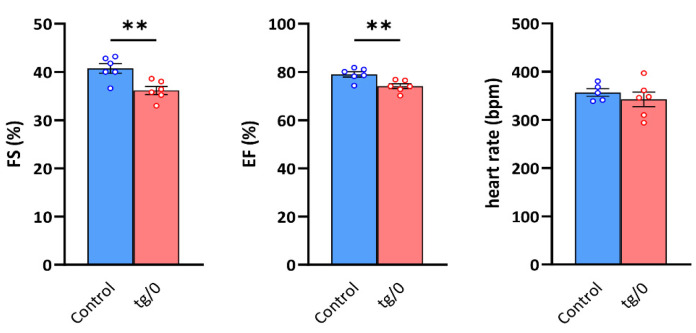
Systolic function of the left ventricle. The function of the hearts in control SHR rats and in SHR-*Tmem70^ko^*^/*ko,tg*/*0*^ (tg/0) animals derived from SHR-*Tmem70^tg^*^/*tg*^ transgenic line 130 were analysed by echocardiography and were compared with the SHR controls. FS—fractional shortening, EF—ejection fraction. Data are mean ± SEM, *n* = 5–6, ** *p* ≤ 0.01.

In the SHR-*Tmem70^ko^*^/*ko,tg*/*0*^ rats, a mild LV systolic dysfunction was detected, manifesting as a decrease in fractional shortening (FS) and ejection fraction (EF) (Figure 8). Other parameters, including heart rate and the geometric dimensions of the LV cavity and wall thicknesses, are presented in Table 1.

### 3.7. Restoration of ATP Synthase Biogenesis

**Figure 9:**
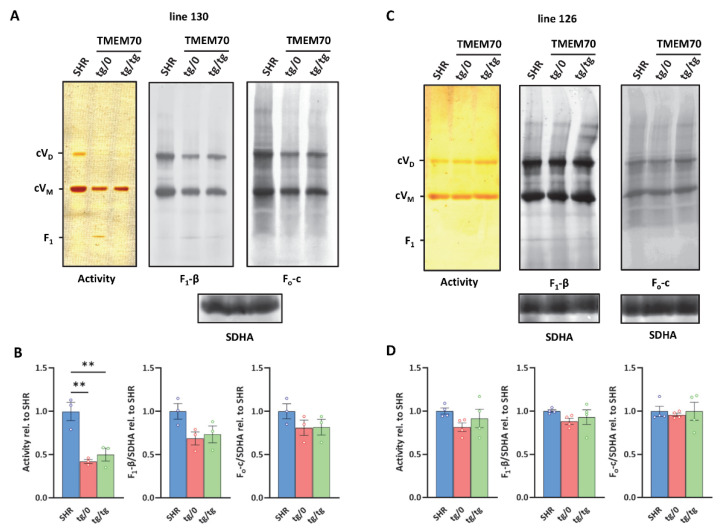
Complementation of ATP synthase biogenesis in the heart. (**A**,**C**) Digitonin-solubilised heart proteins were resolved by blue native electrophoresis (BNE). ATP synthase monomers (cV_M_), dimers (cV_D_), and F_1_ subcomplexes were detected by ATPase activity staining or the immunodetection of F_1_-β or F_o_-c subunits. Complex II content (assessed by SDHA antibody) was used for normalisation. Note that representative images in A for F_1_-β and F_o_-c originate from the same samples on the same membrane developed with different antibodies using two independent fluorescence channels. Therefore, only one SDHA reference is shown. (**B**,**D**) Quantification of the sum of all detected assemblies was performed in control SHR rats and in SHR-*Tmem70^ko^*^/*ko,tg*/*0*^ (tg/0) or SHR-*Tmem70^ko^*^/*ko,tg*/*tg*^ (tg/tg) rats derived from SHR-*Tmem70^tg^*^/*tg*^ transgenic lines 130 and 126. Data are mean ± SEM, *n* = 3–4, ** *p* ≤ 0.01.

All of the structural and functional studies that were performed above convincingly indicated that the transgenic expression of the missing *Tmem70* factor could re-establish ATP synthase content. Since *Tmem70* is involved in F_o_-c incorporation into the holoenzyme, it is feasible to expect that the proper biogenesis of the assembled ATP synthase occurs in *Tmem70*-deficient SHR animals that were rescued by *Tmem70* wild-type expression. Yet, data from the heart suggest that it may (still) be partially compromised in this tissue. Therefore, we analysed the heart samples using native gel electrophoresis (Figure 9) since the observed partial complementation could still leave incomplete and/or aberrant enzyme assemblies, something that is typical of TMEM70 dysfunction [18,21,22].

Heart proteins were solubilized by digitonin and were resolved on blue native electrophoresis (BNE). ATP synthase monomers (cV_M_), dimers (cV_D_), and F_1_ subcomplexes were detected by ATPase activity staining and by means of WB immunodetection of the F_1_-β or F_o_-c subunits (Figure 9A,C). Quantification of the sum of all of the detected assemblies was performed in control SHR rats and in the SHR-*Tmem70^ko^*^/*ko,tg*/*0*^ and SHR-*Tmem70^ko^*^/*ko,tg*/*tg*^ rats derived from the 130 and 126 lines (Figure 9B,D). For the WB data, the signal was normalised to the CII content (using SDHA antibody) that was used as a loading control. Observed Western blot (WB) detection identified the fully assembled ATP synthase complexes in their monomeric and dimeric forms in both the SHR-*Tmem70^ko^*^/*ko,tg*/*0*^ and SHR-*Tmem70^ko^*^/*ko,tg*/*tg*^ rats and in the control SHR rats. Identical expression profiles were obtained using antibodies to the F_1_-β or F_o_-c subunits. Both types of ATP synthase complexes were also visualized by their ATPase hydrolytic activity. In transgenically rescued rats that were derived from line 126, we identified the same functional ATP synthase complex content in the SHR-*Tmem70^ko^*^/*ko,tg*/*0*^, SHR-*Tmem70^ko^*^/*ko,tg*/*tg*^, and control SHR animals (Figure 9D). In case of transgenically rescued rats derived from line 130, the transgenic restoration of ATP synthase biogenesis was less efficient, resulting in the total content of assembled ATP synthase evaluated by the F_1_-β antibody to be 68.6 ± 12.6% of the controls in SHR-*Tmem70^ko^*^/*ko,tg*/*0*^ and 73.5 ± 17% of the controls in the SHR-*Tmem70^ko^*^/*ko,tg*/*tg*^ rats (Figure 9B), albeit not even this decrease reached statistical significance. However, in the case of transgenically rescued rats derived from line 130, we only identified full-size, assembled, and active enzyme complexes. The F_1_ subcomplex of the ATP synthase that typically accumulates in the cells and tissues of patients or animal models that lacks the TMEM70 protein [18,21] was only identifiable in trace amounts in the SHR-*Tmem70^ko^*^/*ko,tg*/*0*^ rats derived from line 130 (2.7 ± 0.15% from the total ATP synthase content evaluated by F_1_-β antibody or 10.2 ± 1.4% of the total signal when assessed by hydrolytic activity staining).

### 3.8. Respiratory Chain Complex I Assembly

**Figure 10:**
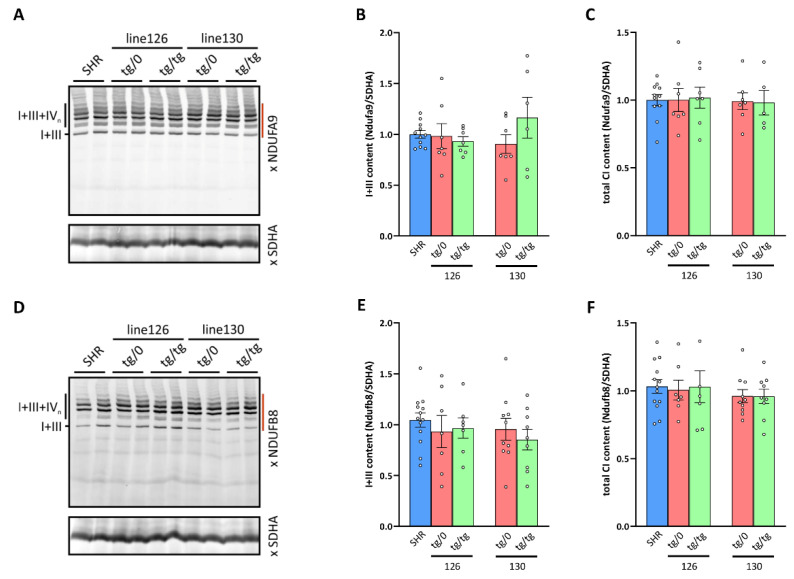
Respiratory chain Complex I biogenesis in the heart. (**A**,**D**) Digitonin-solubilized heart proteins were resolved by BNE electrophoresis and Complex I heterodimer with Complex III (I+III) and Complex I-containing supercomplexes (I+III+IV_n_) were detected by the WB of the Ndufa9 or Ndufb8 subunits. (**B**,**E**) Heterodimer I+III content and (**C**,**F**) the sum of all Complex I assemblies (integrated signal from the area of the gel denoted by dark red line to the left of WB images) normalized to Complex II content (SDHA subunit) were quantified in control SHR rats and in two lines (130 and 126) of derived *Tmem70*^−/−^ knockout rats expressing the *Tmem70* transgene at the single allele SHR-*Tmem70^ko^*^/*ko,tg*/*0*^ (tg/0) or at both alleles SHR-*Tmem70^ko^*^/*ko,tg*/*tg*^ (tg/tg). Data are mean ± SEM, *n* = 5–13.

By measuring the oxygen consumption, we identified the decreased respiration with CI-dependent substrates in the heart from SHR-*Tmem70^ko^*^/*ko,tg*/*0*^ and SHR-*Tmem70^ko^*^/*ko,tg*/*tg*^ animals that were derived from line 130 and 126 (Figure 7A), which brings into question assembly status of CI. Therefore, we used the same approach as above to analyse the biogenesis and assembly of CI (Figure 10). As expected, WB detection with two antibodies to the CI subunits, NDUFA9 (Q module, Figure 10A) and NDUFB8 (P_D_ module, Figure 10D), identified these subunits in the heterodimer of the CI in the control SHR rats with CIII and in the supercomplexes of CI, CIII, and CIV (I+III+IV_n_ – SC) with higher molecular weights, an electrophoretic profile that is typical for the normal biogenesis and assembly of CI. We also observed an identical profile for the hearts from both the SHR-*Tmem70^ko^*^/*ko,tg*/*0*^ and SHR-*Tmem70^ko^*^/*ko,tg*/*tg*^ animals that were derived from lines 126 and 130. Additionally, we also quantified the content of the I+III heterodimer (Figure 10B,E) and the total content of the assembled CI (i.e., CI+III, and SC) in these samples (Figure 10C,F), which revealed the same specific CI content in the SHR-*Tmem70^ko^*^/*ko,tg*/*0*^ and SHR-*Tmem70^ko^*^/*ko,tg*/*tg*^ rats and in the control rats. Therefore, the unaltered biogenesis and a normal content of CI was found in the hearts of ^the^ SHR *Tmem70* knockout rats expressing the *Tmem70* transgene.

## 4. Discussion

The aim of the study was to complement the embryonic lethality of *Tmem70* knockout in the SHR by the transgenic expression of the wild-type *Tmem70* gene. Using this transgenic rescue approach, we explored the potential for gene therapy for neonatal encephalo-cardio-myopathy caused by the dysfunction/absence of TMEM70, a specific assembly factor of the mammalian ATP synthase. This autosomal recessive disorder primarily affects the physiological function of the heart, brain, liver, and other tissues with high energetic demands, although the biogenesis of the ATP synthase and thus mitochondrial energy conversion by the OXPHOS system are impaired in all tissues. Specifically, we questioned whether the transgenic expression of the wild-type *Tmem70* gene can rescue a complete absence of the *Tmem70* factor and to what extent it can complement pathologic phenotypes at organismal and organ-specific levels. We performed our studies in an SHR rat model of essential hypertension and cardiac hypertrophy. Under specific environmental conditions (high fructose/sucrose or folate deficient diets) or after genetic modification (e.g., when expressing resistin or CRP transgenes), SHR animals also develop disturbances associated with metabolic syndrome [53,54,55,56,57,69,70,71,72,73]. Additionally, while these animals progressively develop hypertension and associated comorbidities, they are not severe at a young age. Thus, the phenotypic manifestation of any potential transgene can be evaluated against “healthy” phenotypes as well as against phenotypes recapitulating some aspects of human metabolic syndrome [57,74].

For the transgenic complementation of the TMEM70 defect, we used the Sleeping Beauty (SB) transposon system [75]. To drive the transgenic expression of the *Tmem70* gene, we used the EF-1α promoter, which represents a versatile expression system with a wide host range and that is able to achieve good expression efficiency. Generally, it promotes the robust and constitutive expression of the gene of interest. It has also been tested for gene therapy approaches and has been found to be suitable for both short as well as long/polycistronic transcripts [76]. It has also been previously used in several studies on SHR rats, where it promoted robust transgene expression in the liver but also in the brain, muscle, heart, and kidney [54,77]. Indeed, we also observed analogous tissue patterns in this study, where transgenic *Tmem70* transcripts were detected in all of the tested tissues (Figure 2).

Transgene expression across the broad range of tissues may be critical for the successful complementation of the TMEM70 factor in patients. Universal expression represents an inherent advantage for any potential gene therapy for mitochondrial disorders since they affect multiple organ systems and because the global expression of the transgene is required [78]. In this regard, it has to be kept in mind that successful disease complementation attempts are only able to take advantage of disease progression mitigation by targeting the selected organ. Thus, the *Ndufs4^−^*^/*−*^ animals were rescued by targeting the brain [44,45]; the *Ethe1^−^*^/*−*^ [39] or MNGIE model mice [40,41] were rescued by the hepatotropic AAV2/8 vector; and utility of AAV treatment in the *Ndufs3^−^*^/*−*^ mice was only validated in skeletal muscle-specific knockout [79]. So far, human clinical studies have been limited to LHON patients, where successful delivery to optical nerve should be sufficient for treating the patient [80].

Nevertheless, prospects for genetic complementation may be more optimistic for TMEM70 (or assembly factors in general) since it is present at much lower than stoichiometric proportions relative to the structural subunits of the ATP synthase [18,81]. Our results further indicate that even lower than physiological levels (e.g., 16.3 ± 0.6% of controls in liver of SHR-*Tmem70^ko^*^/*ko,tg*/*0*^ animals derived from line 130) may suffice for the full complementation of ATP synthase content (Figure 4) and function (Figure 6A). Importantly, the residual levels of a functional ATP synthase can assemble both in the *Tmem70^−^*^/*−*^ mouse model [22] and in patients, even in the absence of the TMEM70 protein. These residual levels oscillate around 30%, which is close to the threshold for the onset of pathologic presentation [47]. Therefore, any increase in the assembled ATP synthase content can, at least partially, mitigate the pathologic phenotype in patients. While it is still important to achieve transgenic expression in the majority of tissues, even minuscule TMEM70 expression may be enough for a sufficient outcome. Our results also indicate that even if the transgenic *Tmem70* expression pattern (Figure 2) substantially differs from the physiologic pattern across tissues [22], it is sufficient for successful complementation without any substantial disturbances.

In principle, we achieved better complementation of the ATP synthase defect in the liver than in the heart. In both tissues, the complementation level generally followed transgenic *Tmem70* mRNA expression and hence the TMEM70 protein content that was observed for each individual strain that was analysed. In the case of the liver, we observed somewhat lower translational efficiency for transgenic *Tmem70*. This was manifested in SHR-*Tmem70^ko^*^/*ko,tg*/*tg*^ animals that had been derived from line 126, where the transcript levels were even higher than they were in the controls, yet they still only yielded a TMEM70 protein content of 49 ± 19.5% (relative to controls) in these rats. This indicates that transgenic complementation may not only depend on the tissue specificity of the used promoter, but that it may also depend on regulatory regions in the UTRs of the recombinant construct, which may also confer an additional level of specificity.

Importantly, even a single allele of transgenic *Tmem70* was sufficient to induce the robust renewal of ATP synthase content and biogenesis as well as the restoration of mitochondrial OXPHOS energy provision and enabled full restoration in the liver. In general, the SHR-*Tmem70^ko^*^/*ko,tg*/*0*^ animals that were derived from line 130 demonstrated less effective complementation than the SHR-*Tmem70^ko^*^/*ko,tg*/*0*^ animals that were derived from line 126—this was caused by lower levels of transgenic TMEM70 protein expression in the former line (Figure 4) and corresponds well to the transgenic *Tmem70* expression levels in the parental SHR-*Tmem70^tg^*^/*tg*^ animals. Thus, less than 20% of the SHR wild-type TMEM70 protein led to the full restoration of the physiological functions of the mitochondria in the liver and showed that even single-allele *Tmem70* expression was sufficient for functional complementation. In the case of the heart, the TMEM70 protein content had to reach at least 40% of its control levels in the SHR animals to complement heart function, for which both transgenic alleles were essential. A comparison of the two strains of transgenically rescued animals, the SHR-*Tmem70^ko^*^/*ko,tg*/*tg*^ rats derived from lines 130 and 126 and differing in *Tmem70* expression in both the liver and heart, clearly showed more efficient complementation in the liver compared to in the heart and indicated that the threshold level of TMEM70 protein expression for full complementation in the heart had to be 40% above the control levels.

Furthermore, our study clearly confirmed the direct dependence of ATP synthase subunits content on the restoration of the TMEM70 protein. This is especially true for F_o_-c subunit, which is critical for the proper assembly of the ATP synthase [10] and represents the direct target of TMEM70 action. At the level of respiratory chain function, full complementation of the ATP synthase also normalized OXPHOS function, while insufficient complementation of the ATP synthase content limited ATP production, as indicated by a decrease in the RCR. Interestingly, it also decreased the electron transport capacity of the respiratory chain, as revealed by lower rate of the state 3-uncoupled oxidation of both the FADH_2_- and NADH-dependent substrates. In this regard, it is interesting that TMEM70 was found to be associated with Complex I assembly factors on the complexomes [82] and that its knockout in HAP-1 cells affected the stability of the membrane-bound Complex I subassemblies [24]. Complex I deficiency is also a rather frequently (almost 50%) observed secondary defect in muscle biopsies from TMEM70-deficient patients [34]. However, no drop in the content of Complex I subunits was observable by LFQ quantification in the mice in a *Tmem70^−^*^/*−*^ knockout model [22], and the assembled Complex I content was not changed in the fibroblasts of patients with the most common c.317-2A>G homozygous mutation [83]. Indeed, in our case of hearts expressing the *Tmem70* transgene, neither the content of the representative Complex I subunits (Figure 5B) nor the content of assembled Complex I (Figure 10) were affected. Since we also observed a drop in the content of the Complex III subunits (Figure 5B), it is possible that the observed decrease in uncoupled respiration was the result of a general energetic failure (insufficiency) rather than a specific dysfunction in respiratory chain Complex I. Nevertheless, the role of *Tmem70* in the biogenesis of Complex I still warrants further investigation.

To evaluate cardiac function in vivo, we only focussed on the animals who were the most biochemically affected (SHR-*Tmem70^ko^*^/*ko,tg*/*0*^ rats derived from line 130), which were also leaner than the controls. Indeed, in these animals, we determined reduced LV systolic function, but this drop was rather mild. Interestingly, this matches well the observed decrease in the fractional shortening seen in mice that were heterozygous for *Tmem70* knockout (*Tmem70^+^*^/*−*^*)* [21] as well as in the SHR-*Tmem70^ko^*^/*0*^ rats [58]. Theoretically, these rodent knockout heterozygotes would be phenotypically similar to TMEM70^+/−^ carriers in the human population, i.e., the parents of TMEM70 patients. However, no functional data are available for human pathology in this regard.

## 5. Conclusions

We have demonstrated that the transgenic expression of *Tmem70* rescued embryonic lethality in SHR model and that ATP synthase defects can be amenable to gene therapy. Our results show that even low levels of the transgenic TMEM70 protein may warrant successful complementation, which is an important prerequisite for future consideration in human medicine. In addition, the SHR-*Tmem70^ko^*^/*ko,tg*/*0*^ animals that were derived from the SHR-*Tmem70^tg^*^/*tg*^ 130 transgenic line also represent the first rodent model of *Tmem70*-associated heart pathology, which may be useful in future animal studies of *Tmem70* function in the heart.

## Figures and Tables

**Table 1:** Geometry of the left ventricle and heart rate in the control SHR and SHR-*Tmem70^ko^*^/*ko,tg*/*0*^ rats.

	Control	Tg/0
AWTd (mm)	1.89 ± 0.05	1.79 ± 0.04
LVDd (mm)	7.50 ± 0.16	7.38 ± 0.18
PWTd (mm)	1.86 ± 0.06	1.76 ± 0.04
AWTs (mm)	2.86 ± 0.05	2.62 ± 0.02 *
LVDs (mm)	4.46 ± 0.15	4.71 ± 0.16
PWTs (mm)	2.93 ± 0.10	2.65 ± 0.04 *
HR (b.p.m.)	339 ± 17	343 ± 14

AWTd, diastolic anterior wall thickness; LVDd, diastolic left ventricle diameter; PWTd, diastolic posterior wall thickness; AWTs, systolic anterior wall thickness; LVDs, systolic left ventricle diameter; PWTs, systolic posterior wall thickness; HR, heart rate; b.p.m., beats per minute. Values are means ± SEM from six rats in each group. * *p* < 0.05 vs. SHR control.
